# Adaptation of the Vaccine Prophylaxis Strategy to Variants of the SARS-CoV-2 Virus

**DOI:** 10.3390/vaccines13070761

**Published:** 2025-07-17

**Authors:** Sofia M. Gulova, Uliana S. Veselkina, Irina V. Astrakhantseva

**Affiliations:** Department of Immunobiology and Biomedicine, Sirius University of Science and Technology, 354349 Sirius, Krasnodarsky Krai, Russiauliana.veselkina@talantiuspeh.ru (U.S.V.)

**Keywords:** SARS-CoV-2, vaccine, viral evolution, pan-coronavirus vaccine, viral variants, immune evasion

## Abstract

The emergence of a novel severe acute respiratory syndrome coronavirus 2 (SARS-CoV-2) virus closely related to SARS-CoV and officially known as *Betacoronavirus pandemicum* precipitated a substantial surge in vaccine development that culminated during the global COVID-19 pandemic. At present, there are dozens of vaccines for the prevention of SARS-CoV-2 being utilized across the globe. However, only 10 of these vaccines have been authorized by the World Health Organization (WHO). These include mRNA-based, viral vector, subunit and whole-virion inactivated vaccines. At the current end of the pandemic, there has been a decline in the global vaccination rate, both for the general population and for those most at risk of severe illness from the virus. This suggests that the effectiveness of the vaccines may be waning. The decline occurs alongside a decrease in testing and sequencing for SARS-CoV-2. Furthermore, the process of tracking viruses becomes increasingly complex, thereby providing a selective advantage for SARS-CoV-2 and allowing it to evolve stealthily. In this review, we provide a comprehensive overview of viral evolution and vaccine development. We also discuss ways to overcome viral variability and test universal vaccines for all SARS-CoV-2 variants.

## 1. Introduction

The emergence of the novel SARS-CoV-2 virus, which is closely related to the SARS-CoV virus and officially known as *Betacoronavirus pandemium*, caused a global pandemic in 2019 [1,2]. This event has led many research institutions and biotechnology companies to develop different vaccines. These vaccines differ in the platforms on which they are based and their effectiveness [3,4].

The viral protein of SARS-CoV-2 responsible for the cell entry process via ACE2 receptor is the spike (S) protein, which is a target for neutralizing antibodies and is the main protein for the vaccine design [5,6,7,8]. The S protein also contains a variety of epitopes that are recognized by CD4+ and CD8+ T cells, and, generally, is more immunogenic compared to other SARS-CoV-2 proteins [9]. Consequently, that protein is under constant evolutionary pressure. It should also be noted that the evolution of SARS-CoV-2, resulting in variants that effectively evade the immune response, almost always involves a mutation in the S protein. Despite the initiation of a widespread vaccination campaign against the SARS-CoV-2 a year after its identification, mutations in the pathogen have been identified and have eventually led to new waves of infection [10]. It is also evident that prior to the emergence of Omicron, the reinfection rate remained low, and the number of circulating variants was found to be limited in comparison to the number of new cases. However, the Omicron-era high reinfection rate could be indicative of the transmissibility and immune evasion capabilities of Omicron. It is suggestive of its capacity to circumvent both natural immunity and vaccine-induced protection [11].

As most authorized vaccines target the production of neutralizing antibodies against the S protein to prevent infection with the SARS-CoV-2 virus, multivalent or updated vaccines have been suggested with the introduction of new variants including those of the Omicron family [12,13,14,15].

In the present moment, we find ourselves amid the onset of the low epidemic state of SARS-CoV-2 [16]. However, despite a decline in the number of new cases of infection, dominant variants are changing rapidly, approximately every two to three months, reflecting the ongoing strong competition within the viral population [17]. Consequently, there is an urgent need for a re-evaluation of current vaccination strategies and regulatory frameworks.

The present review aims to draw attention to the significant period of evolution of SARS-CoV-2 virus, and to consider the factors that have led to the erosion of the vaccine’s effectiveness. In addition to this, the current state of research in the field of universal vaccine design will be summarized.

## 2. Pre-Omicron Era: The Race Between Vaccine Development and SARS-CoV-2 Evolution

The majority of vaccines against COVID-19 use S protein as main immune-inductive agent since it is highly immunogenic thanks to a glycoprotein nature. However, new variants of SARS-CoV-2 are usually distinguished by specific S protein mutations. Additionally a number of bioinformatic articles describe changes in other structural proteins, and these changes should also influence viral properties as well as vaccine effectiveness [18,19].

The first significant mutation of the SARS-CoV-2 virus was D614G located in the B cell epitope of the S1 subunit of S protein. That mutation is now one of the main variants utilized in studies as a reference for the pre-Omicron era. The D614G mutation first appeared in April 2020 and rapidly became the most prevalent form within the viral population [20,21]. It has been demonstrated that the G614 S protein exhibited enhanced binding affinity to the ACE2 receptor. Furthermore, it was observed to elevate viral loads within the respiratory tract, thereby enhancing its transmissibility. However, no significant change in susceptibility for the neutralization of a virus was observed [22,23,24]. So, G614 did not drastically mitigate efficacy of the first developed vaccines based on the Wuhan variant [20,22].

Among all pre-Omicron variants, the Alpha (lineage B.1.1.7), Gamma (lineage P.1), Beta (lineage B.1.351) and Delta (lineage B.1.617.2) variants caused the most concern [25]. It was demonstrated that all of the variants exhibited a greater degree of affinity for the ACE2 receptor in comparison to the D614G variant (see all significant viral changes in Table 1) [26].

The Alpha variant was the first variant of concern (VOC) following the emergence of D614G in October 2020. This variant was first identified in the United Kingdom, and its key mutation, N501Y, was located in the RBD [27]. The mutation in this variant led to significantly increased transmissibility and fusogenicity [26]. That variant had been persisting mostly in Europe so was thoroughly examined. Despite the enhanced transmissibility of Alpha variant and more severe disease outcome, it was not able to escape from neutralizing antibody (nAb) activity elicited by vaccines or natural infection [28,29,30,31,32].

**Table 1:** The key mutations of SARS-CoV-2 in more prevalent variants.

Variant	Date of Emergence	Key Mutation	Reference	Key Features
G614	April 2020	D614G *	[23,33]	Enhanced viral replication by increased infectivity and stability of virion-ACE2 interaction
Alpha (B.1.1.7)	October 2020 (UK)	D614G, N501Y, **P681H,** R203K, G204R, D1118H, ΔH69/V70, *∆Y144* (***+IE***), A570D	[26,27,34,35]	Higher viral load Increased infectivity and infectiousness Improved binding to ACE2 receptor Enhanced S protein cleavage
Beta (B.1.351)	September 2020 (South Africa)	D614G, D215G, *K417N* (***+IE***), E484K, N501Y	[36]	Enhanced transmissibility Resistance to neutralizing antibodies
Gamma (P.1)	November 2020 (Brazil)	D614G, N501Y, E484K, K417T	[37,38]	Increased RBD affinity to ACE2 Increased transmissibility and virulence Immune evasion
Delta (B.1.617.2)	October 2020 (India)	D614G, L452R, T478K, **P681R**	[37,39,40]	Increased fusogenicity and infectivity 60% more transmissible than the Alpha variant Improved furin site recognition by proteases
Omicron BA.1 (B.1.1.529.1)	November 2021 (South Africa)	D614G, E484A, N501Y, Q493K, Q498R, *K417N*, S477N, ***Y505H***, G496S, ***T478K***, L452R S375F, S371L	[39,41,42,43,44]	Diminished fusogenicity, pathogenicity and cleavage efficacy (relative to Delta variant) Heightened transmissibility and infectivity Higher reinfection possibility Immune evasion
BA.2 (B.1.1.529.2)	November 2021 (India)	BA.1+ ***T19I***, ∆PPA25–27, ***G142D***, V231G, S371F, T376A, D405N, R408S	[45,46,47]	Increased transmissibility Improved fusogenicity Better S protein cleavage and ACE2 affinity (compared to BA.1)
XBB	August 2022 (India)	*∆Y144* (***+IE***), **P681H** V83A (***+IE***), H146Q, ***Q183E***, V213E, R346T (***+IE***), N460K G339H, ***R368I***, ***V445P***, ***G446S***, ***F490S***, *F486S* (***+IE***)	[48,49,50]	Increasing its fitness through recombination rather than substitutions. (recombination of BJ.1 and BM.1.1.1) Improved transmissibility and Enhanced immune evasion (relative to BA.1 Omicron)
XBB.1	August 2022 (India)	XBB+ *G252V*	[49]	Greater fusogenicity (in comparison to BA.2.75) Profound resistance to antiviral humoral immunity induced by prior Omicron subvariants
XBB.1.5—Kraken	December 2022	XBB.1+ S486P	[51]	Enhanced binding affinity to ACE2 receptor (compared to XBB.1) Increased transmissibility Immune evasion capabilities are the same as XBB.1
BA.2.86	July 2023 (Israel, Denmark)	*∆Y144* (***+IE***) F157S, **P681R**, G339H, N460K, F486P, ***A484K***, *L452W* (***+IE***)***,*** *A445H* (***+IE***), ***N450D***, *∆*N211, A264D, *S50L*, *L216F*, *K356T* (***+IE***), *R403K*	[52,53]	Substantial antigenic drift Enhanced receptor affinity
JN.1	September 2023 (India)	BA.2.86+ L455S	[54,55]	Significantly improved fusogenicity Increased infectivity
SLip		JN.1+ *F456L* (Flip mutation)	[56,57]	Decreased infectivity and membrane fusion Declined spike processing compared to JN.1 Immune evasion
FLiRT		SLip+ R346T	[57]	Immune evasion Decreased infectivity, cell-cell fusion, and spike processing relative to JN.1
KP.2	January 2024	Flirt+ V1140L	[58]	Immune evasion Decreased infectivity, cell-cell fusion, and spike processing relative to JN.1
HK.3	January 2023 (East Asia)	EG.5.1+ L455F (Flip mutation)	[54]	Enhanced immune evasion Increased fusogenicity (compared with EG.5.1)
KP.3	February 2024	JN.1+ Q493E	[59,60,61]	Diminished infectivity and affinity to ACE2 Immune evasion
KP.3.1.1	March 2024	KP.3+ ΔS31	[61]	KP.3, KP.3.1.1 and XEC showed a significant increase in ACE2-Spike binding affinity compared with JN.1; no significant changes in the receptor binding of KP.3.1.1 and XEC relative to KP.3
XEC	June 2024	KP.3+ F59S, T22N	[61,62]	Enhanced binding affinity to ACE2 receptor in comparison to JN.1, but not to KP.3 Reduced cell–cell fusion relative to its parental KP.3

* improvement in cleavage, improvement in affinity/fusogenicity, *decrease in affinity/fusogenicity*. IE—immune evasion.

In September 2020, a novel variant was identified in South Africa. This variant was subsequently designated as Beta (B.1.351) and possessed five additional mutations in the S protein: D80A, D215G, E484K, N501Y and A701V. Subsequent to this identification, three further mutations were identified in November 2020, L18F, R246I and K417N, in conjunction with a deletion at position 242 to 244. This lineage underwent rapid proliferation, ultimately achieving a predominant status within South Africa [63]. The literature regarding the efficacy of mRNA vaccines in combating the Beta variant has yielded conflicting results. One study has documented a substantial decline in the neutralization titer of vaccine-induced antibodies against the Beta variant in sera from individuals vaccinated with the mRNA-1273 (Moderna) and BNT162b2 (Pfizer BioNTech) vaccines [64]. The other studies have shown that mRNA vaccines demonstrated an efficacy against the Beta variant equivalent to that against the Alpha variant [65,66]. A significant decrease in the neutralizing effectiveness of inactivated whole-virion, vector and subunit recombinant vaccines was observed [28,31,32]. For instance, subunit vaccine NVX-CoV2373 (Novavax) has demonstrated drastically reduced efficacy against the Beta variant relative to the Alpha variant (49,4% and 85,6% respectively) [32]. Furthermore, the clinical trial for the ChAdOx1 nCoV-19 vaccine (Oxford-AstraZeneca) did not demonstrate protection against mild to moderate cases of SARS-CoV-2 due to the B.1.351 variant [67,68].

The emergence of the Gamma variant at the end of 2020 in Brazil is of particular interest. This variant harbors 17 mutations; 10 of them were located in the S protein and displayed significantly increased transmissibility compared to the other local viral variants, higher pathogenicity independent of sex and age and increased lethality [69,70]. The mRNA vaccines (mRNA-1273 and BNT162b2) had failed to protect against the Gamma variant. In contrast, studies conducted on the whole-virion inactivated vaccine CoronoVac (by Sinovac Biotech) have indicated an efficacy rate of approximately 50% [70,71]. Notably, the ChAdOx1 nCoV-19 vaccine of Oxford-AstraZeneca demonstrated a 77.9% efficiency against the Gamma variant, which is the highest among all other vaccines [72].

Despite the variable neutralizing abilities of antibodies induced by vaccine against early variants of SARS-CoV-2, the analysis of CD8+ T cells response in convalescent individuals of the North American population has revealed conserved CD8 epitopes non-affected by mutation [73].

The Delta variant first appeared in India in October 2020 and then spread rapidly around the world. It showed extremely increased transmissibility and lethality compared to previous VOCs [26,74]. It had overtaken other VOCs and persisted in populations for a longer period of time, as all mutations (in both N-terminal domain (NTD) and RBD, the most notable being L452R, E484Q and P681R) contributed to the increased fitness of the variant [75,76]. The S protein vaccines such as BNT162b2, ChAdOx1 nCoV-19 and Ad.26.COV.2.S (Janssen/Johnson & Johnson) showed reduced efficacy against Delta variant compared to the Alpha, but still provided protection; however, breakthrough infections were observed [77,78]. Whole-virion inactivated vaccines, for example, Covaxin (Bharat Biotech), displayed lowered efficiency against VOCs, yet still provided robust immune memory with a modest impact on the humoral response against Delta and Gamma variant [79]. The indisputable advantage of the whole-virion inactivated vaccines over other types is a wide variety of induced antibodies (which, overall, should increase protection against virus) as well as broad B- and T-cell response [79]. However, a lot of whole-virion vaccines are now discontinued as they have shown low efficiency against Omicron variants and were not updated. Recombinant S protein vaccine SCB-2019 (Clover Biopharmaceuticals) is of particular interest since it has displayed increased efficacy against Delta variant—79%, compared to 67,2% against the initial Wuhan variant [13]. Overall, recombinant vaccines showed reduced efficacy against the Delta variant; however, sera from vaccinated individuals demonstrated neutralizing activity higher than that of convalescent sera [13]. The studies reviewed in [9] have demonstrated that CD4+ and CD8+ T-cell responses induced by SARS-CoV-2 infection or vaccination with various types of vaccine are able to recognize all circulating SARS-CoV-2 variants at that time, regardless of geographic location.

Other VOCs, including Epsilon, Zeta, Eta, Theta, Iota, Kappa, Lambda and Mu, appeared in late 2020 and the first half of 2021. These variants are characterized by various changes in viral properties, but all were eventually replaced by the Delta variant [76,80].

## 3. Vaccine Efficiency in the Omicron Landscape

The substantial evolutionary progression of the SARS-CoV-2 virus was marked by the emergence of the Omicron variant in late 2021 in South Africa, a development that occurred as a consequence of recombination and an antigenic shift [27,35]. One of the main concerns was the propensity of Omicron to reinfection. At the same time, Omicron variants are associated with a significantly reduced pathogenicity and hospitalization rate due to the lowered fusogenicity of their S protein [27].

The emergence of the BA.1 and BA.2 variants was precipitated by mutations in the Omicron lineage. As can be deduced from the available data, both have approximately 30 mutations in their spike proteins. These mutations result in increased transmissibility and increased fusogenecity, with the consequent effect of a reduction in the number of severe cases (Table 1) [76]. Both variants strongly resist neutralization with the sera of people immunized with any type of WT vaccine [81,82,83]. Starting from these variants, a three-dose vaccination schedule was recommended for better protection [84]. Two bivalent boosters have been developed, each containing the BA.1 variant. These boosters were derived from the BNT162b2 and mRNA-1273 vaccines, and both have been shown to elicit a robust humoral and T cell response against BA.1, BA.2 and the later-emerging BA.4/BA.5 variants [85]. Notably, three doses of NVX-CoV2373 vaccine elicited responses similar to mRNA vaccines and provided protection against BA.4/5 variants as well [86].

The XBB variant first emerged in India in 2022 as a result of recombination between two variants of the BA.2 sublineages. XBB sublineages have reduced antigenicity compared to BA.2.86 but slightly increased antigenicity compared to pre-XBB Omicron variants [87,88,89]. It is imperative to note that key point mutations of XBB sublineages are R346T and G252V and they provide partial S309 monoclonal antibody (mAb) evasion; S486P mutation of XBB.1.5 variant enhances binding to ACE2 receptor and increases the antigenicity of the virus. Finally, the L455F and F456L mutations contributed to the emergence of the FLip variant and have also been observed as mutation spots in the JN.1 lineage [57,88]. Interestingly, no mutations of XBB subvariants resulted in complete evasion from S309 mAb, in contrast to the BA.2.86 sublineage, which demonstrated robust resistance towards S309 therapy [87,88]. A bivalent mRNA vaccine containing WT and BA.4/5 S protein (Pfizer BioNTech) had proved its efficacy against the XBB.1 variant (G252V mutation) [90,91]. Further XBB subvariants exhibited no significant resistance to neutralization in a cohort vaccinated with three doses of the WT mRNA vaccine. As expected, neutralization was better in the cohort that showed breakthrough infection and the effect was demonstrated not only against XBB subvariants but also BA.2.86 [89]. Yet, still, Xun Wang et al. observed that vaccination with a quadrivalent peptide booster SCTV01E (SinocellTech), which contains BA.1 S protein, provided a neutralizing antibodies (nAbs) titer that was even higher relative to the breakthrough infection group [89]. What is more, SCTV01E provided cross-protectivity against both BA.2.86 and XBB sublineages [89,92]. Currently, this vaccine is undergoing phase III clinical trials and has been approved for emergency use in China [92]. A paucity of data exists regarding the efficiency of SCTV01E when employed against the contemporary variant of interest (VOI)—JN.1 and its subvariants. However, the XBB.1.5 mRNA monovalent vaccine booster by Moderna and Pfizer BioNTech is more accessible on the market and has proved its efficacy against XBB sublineages and BA.2.86 [87,88,93]. This vaccine demonstrated advantages over the bivalent BA.4/5 mRNA vaccine in terms of the neutralization of BA.2.86 and XBB sublineages since the titers of nAbs in human sera were higher [88,94]. It is important to note that all studies conducted during the circulation of the XBB sublineages and the BA.2.86 variant worldwide had demonstrated a robust immune response to the WT SARS-CoV-2 and D614G strain [87,88,89,90,93]. Novavax has also released the vaccine NVX-CoV2601, updated for the XBB.1.5 variant, which was proven to be effective and safe, and demonstrated cross-reactivity against late Omicron variants such as JN.1 and BA.2.86 [95]. These examples, once again, clearly demonstrate how good vaccine formulation design may possibly impact the population immunity and the importance of updating vaccines against the SARS-CoV-2 virus.

The BA.2.86 variant emerged in the summer 2023 and successfully co-circulated with the XBB lineages. The BA.2.86 S protein was highly mutated compared to its ancestor BA.2 or XBB lineages, with a total mutation count exceeding 30 (Table 1) [87]. BA.2.86 had demonstrated a lack of resistance to neutralization in the sera of individuals who had received a bivalent mRNA vaccine booster (containing BA.4/5 S protein), similar to XBB variants [87]. In contrast, BA.2.86 harbors S protein mutations attributed to increased pathogenicity and displayed a complete evasion from S309 mAb, a trait that is not shared by XBB.1 and XBB.1.5 [87]. Nesamari et al. studied the T-cell response to different viral variants in a group of medical workers from South Africa who had hybrid immunity against SARS-CoV-2 as they previously been vaccinated and infected. The study showed that the S-specific CD4+ T-cell response was highly preserved (≥90%) against the S protein of the ancestral and the Omicron variants, including BA.1, XBB.1 and even BA.2.86. However, the preservation of CD8+ T-cell responses was more variable across the individuals [96]. Further mutation of BA.2.86 in the L455 residue led to the current VOI—JN.1, which completely took over the XBB sublineages [97].

As mentioned before, L455 and F456 residues of RBD have become key mutation spots not only for FLip variants of XBB lineage but also for JN.1 sublineages. JN.1 variant emerged in late 2023 and harbors single S protein mutation—L455S, relative to its ancestor—BA.2.86 (Table 1). The L455S mutation in JN.1 had been shown to reduce its binding affinity to the human ACE2 receptor compared to BA.2.86 [57,98]. Subsequent variants of JN.1, such as Slip (L455S, F456L), FLiRT (R346T) and KP.2 (V1140L), demonstrated even lower infectivity and cell–cell fusion activity, which, overall, reduce viral fitness [57,94,98]. However, these mutations have created significant opportunities for SARS-CoV-2 to evade the immune system, enabling it to spread on a global scale. As of today, the JN.1 variant and its FLiRT subvariant prevail above others [99]. Research groups had investigated JN.1 for nAbs in vaccinated humans and immunized hamsters. It was shown that the bivalent BA.4/5 mRNA vaccine, given together with the WT mRNA vaccine, generates a pool of nAbs for the XBB.1.5 and JN.1 lineages. However, for the JN.1 variant line, the NT50 is reduced [57,94]. There are also data showing an increase in JN.1 variants nAbs titers when a monovalent XBB.1.5 vaccine booster is taken. It is concordant with in vivo studies, which demonstrated robust neutralization against FLip, HK.3 (L455F) and JN.1 subvariants, including FLiRT, SLip and KP.2 with XBB.1.5-monovalent immunized hamster sera, albeit with a reduced efficiency [57,94,100]. Altogether, it proves the necessity of including JN.1 S protein in upcoming vaccine formulations. Currently, there are two JN.1 updated mRNA monovalent vaccines: Bretamoveran by Moderna and Pfizer-BioNTech and the JN.1 vaccine booster by Japanese pharmaceutical company Daiichi-Sankyo. Both treatments induce immunity against JN.1 and provide cross-protectivity for BA.5 and XBB sublineages [101,102]. Interestingly, according to the study by C. Happle et al., Bretamoveran did not appear to be effective for the current variant under monitoring (VUM)—KP.3— whereas K. Uriu et al. reported robust neutralizing activity for KP.3 after a booster. In this study, Bretamoveran shows strong neutralizing activity for KP.3.1.1 and XEC variants (VUMs on February 2025), but Daiichi-Sankyo’s JN.1 booster showed better results. It is important to note the significant differences between the two cohorts of participants in the K. Uriu study in terms of age, sex and previous vaccination status, as these factors may significantly influence the results of the study. One of the key points of both studies is the shift in immunological imprinting towards Omicron variants. This suggests the need to update further vaccine formulations [101,102].

The whole-virion inactivated vaccine has demonstrated its capacity to offer protection against the SARS-CoV-2 ancestral Wuhan variant and other VOCs. This finding signifies its potential for cross-variant protection. However, the vaccine’s effectiveness in preventing infection was significantly lower than that of biotechnology vaccines [103,104,105,106,107]. The emergence of vaccine-evasive variants, such as Omicron lineages, has been accompanied by a noted reduction in neutralization efficacy [79,103,108,109,110]. A proposal has been made to update the whole-virion vaccine, but this appears to be challenging due to the intricate nature of the process and the low yield of production of Omicron subvariants. Notwithstanding the substantial decline in efficacy in preventing infection by SARS-CoV-2, the whole-virion vaccine has demonstrated the capacity to broaden and enhance the quality of SARS-CoV-2-specific immunity, including cellular immunity, and highlights the role of non-neutralizing antibodies in viral elimination [111,112].

As noted above, the Omicron variants do indeed form a distinct phylogenetic clade, and as evolution continues, different Omicron lineages accumulate genomic changes to form another genetically and phenotypically distinct lineage. For example, the characteristics of the BA.2.86 lineage differ significantly from those of the XBB lineage. However, both lineages continue to circulate around the world. Perhaps, subsequent studies and observations on SARS-CoV-2 will shed light on whether it would be advisable to apply serotypes to SARS-CoV-2.

## 4. Prospects for Pan-Coronaviral Antivirals: The S2 Subunit in the Spotlight

The shift in immune imprinting towards Omicron, combined with the antigenic drift of SARS-CoV-2 variants, has led to a decrease in vaccine efficacy against SARS-CoV-2 infection, which could potentially compromise public health. Consequently, there has been a surge in research and development efforts to engineer a next generation of SARS-CoV-2 vaccines that exhibit enhanced breadth and effectiveness in confronting the infection. Morens et al. posit that a pan-coronavirus vaccine should possess the following five ideal properties: (1) the prevention of SARS-CoV-2 infections and breakthrough infections, (2) the induction of long-term mucosal and systemic immunity, (3) the prevention of community transmission, (4) the establishment of durable herd immunity and (5) the provision of universal coverage of Betacoronaviruses [113]. To induce a broad response, antibodies will need to target conserved regions of the S protein. Epitope mapping of broad nAbs identified a class of such antibodies targeting the stem-helix bundle of the S2 in prefusion S trimers that are predicted to disrupt S-mediated membrane fusion. Critically, these antibodies cross-neutralize the human-infecting Betacoronaviruses, including SARS-CoV-2, SARS-CoV, MERS-CoV and human coronaviruses (HCoVs) HKU1 and OC43. Notably, these stem helix-targeting broad nAbs failed to neutralize the Alphacoronaviruses NL63 and 229E [114,115,116,117].

**Figure 1:**
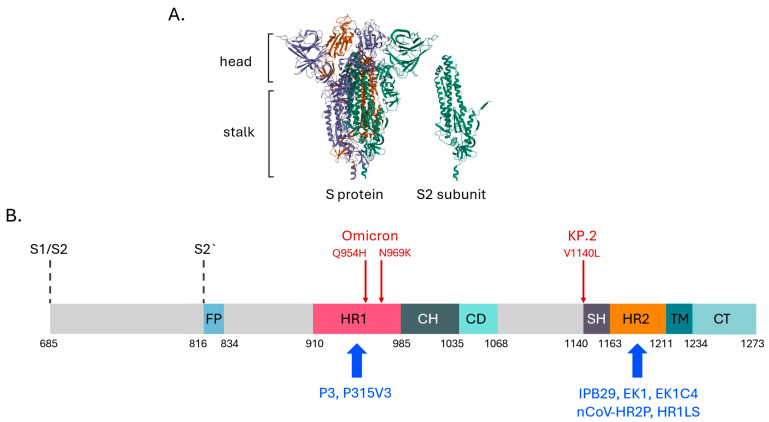
The schematic of the S2 structure of the SARS-CoV-2 S protein. (**A**). 3D Structure of the SARS-CoV-2 S protein on the left; monomeric S2 subunit in the prefusion state on the right. 3D protein structure visualization was obtained using the RSCB Protein Data Bank; data for 8VKK were used to create this figure (**B**). Domain arrangement of S2 subunit of SARS-CoV-2 S protein. S1/S2—furin cleavage site between S1 and S2 domains of the spike protein; S2′—protease furin cleavage site; FP—fusion peptide; HR1—heptad repeats 1; CH—central helices; CD—connector domain; SH—stem helix; HR2—heptad repeats 2; TM -transmembrane domain; CT—cytoplasmic tail. Inhibitory pan-coronavirus agents reviewed in the text are depicted in bright blue; hallmark SARS-CoV-2 mutations featured in the text are depicted in red [122,123,124,125,126].

The S2 subunit of the S protein plays a crucial role in the virus’s internalization process. This process occurs after the RBD binds to the ACE2 receptor [34,118,119]. The S2 subunit consist of regions that are essential for the overall stability of the S protein and that makes the S2 subunit a highly conserved region of the S protein (Figure 1) [34]. It shares up to 90% homology with SARS-CoV with a 100% match for the heptad repeat 2 (HR2) region of SARS-CoV Tor2 strain and MERS-CoV [120]. All those facts make it a prime candidate for further vaccine development. The use of the S2 subunit is intended to generate broadly neutralizing antibodies (bnAbs) that provide protection against multiple variants of SARS-CoV-2 and other Betacoronaviruses [117,121]. For instance, several studies have found cross-reactive antibodies in patients without prior exposure to SARS-CoV-2. These antibodies can efficiently neutralize SARS-CoV-2, although with a reduced efficiency, and have demonstrated binding with the S protein, but not with the S1 subunit alone [117,121]. Similar findings were demonstrated for the T-cell immune response. Memory CD4+ T-cell lines were found to be able to react with SARS-CoV-2. It is noteworthy that there is a positive correlation between the sequence similarity between SARS-CoV-2 and other coronaviruses and the extent of cross-reactivity of CD4+ T cells [121]. The induction of CD4+ T cells is crucial in the development of the vaccine, as T cells have been shown to provide long-lasting protection. In contrast, the levels of nAbs have been observed to decline over a period of several months [121].

In the pre-Omicron era of the pandemic, utilizing the LIBRA-seq approach on a peripheral blood mononuclear cell (PBMC) sample from a donor who was infected with SARS-CoV more than 10 years before the samples were collected, several antibodies were identified. These antibodies bind not only to the S protein of SARS-CoV and SARS-CoV-2 but also to the S proteins of the endemic HCoV-OC43 and HCoV-HKU1, but at lower levels. It was observed that these antibodies targeted cross-reactive epitopes on S2 but could not neutralize SARS-CoV or SARS-CoV-2. However, two of the antibodies showed an ability to cause cross-coronavirus phagocytosis, and four antibodies were found to be capable of mediating trogocytosis. Additionally, two of these antibodies were tested in vivo and exhibited a reduction in lung hemorrhage score, although the impact on viral load remained inconclusive [127]. The 86% of individuals infected with 229E and OC43 coronaviruses exhibited IgG against the S2 subunit of the S protein of SARS-CoV-2 [128]. Additionally, it has been demonstrated that S2-reactive CD4+ T cells with a predominantly Th1 memory phenotype are present in seronegative SARS-CoV-2-unexposed donors and S2 T-cell epitopes have been shown to exhibit a higher degree of similarity to the human endemic coronaviruses 229E, NL63, OC43 and HKU1 with regard to the SARS-CoV MHC-II epitopes, in comparison to the S1 T-cell epitopes [129]. In the other cohort of samples also collected prior to the emergence of SARS-CoV-2 (2011 to 2019), which encompassed healthy donors, pregnant women and young, healthy donors, a range of 3 to 43% exhibited detectable levels of SARS-CoV-2 S protein cross-reactive IgG antibodies, accompanied by the presence of virus-neutralizing activities [130]. The infection of SARS-CoV-2 resulted in a significant increase in the circulation of cross-specific antibodies for the closely related HCoVs. A depletion experiment with soluble monomeric SARS-CoV-2 S1 or S2 subdomains indicates that S2-specific antibodies have a greater contribution to cross-reactivity than S1-specific antibodies, which is in accordance with the higher conservation of the S2 subdomain compared to the S1 subdomain [131]. The discovery of monoclonal antibodies specific to the S2′ cleavage site, which is essential for viral entry into the host cell, was soon made (COV44–62 and COV44–79). These antibodies were also found to be capable of neutralizing Alpha- and Betacoronaviruses, including the Omicron subvariants BA.2 and BA.4/5 of SARS-CoV-2 [118]. However, their potency was determined to be lower compared to RBD-specific antibodies [115]. The other anti-S2 mAb CV804 demonstrated binding activity against multiple variants, including various Omicron subvariants (BA.2.75, BA.4.1, BA.4.6, BE.1, BA.5.2.1, BQ.1.1, XBB.1, XE, BF.7 and BF.7.4.1); however it was not able to neutralize SARS-CoV-2 in a virus neutralization test. In addition, its therapeutic effect in mouse SARS-CoV-2 infection was also limited [132]. It is important to note that the antibody response to linear peptides derived from conserved areas of the SARS-CoV-2 virus in the S2 C-terminal region is significantly increased in infected individuals when compared to vaccinated individuals [133].

The S2 subunit serves as a stalk of spike protein and includes multiple α-helical structures (Figure 1B) [119]. Altogether, it forms the machinery essential for the viral entry to the cell. The formation of the six-helix bundle (6-HB) by the interaction between HR1 and HR2 domains brings viral and cellular membranes together for subsequent fusion, resulting in viral entry into host cells. The Q954H and N969K mutations, which are present in the HR1 domain of all Omicron subvariants (Figure 1B), do not appear to be involved in the HR1–HR2 interaction, suggesting that these HR1 mutations have a minimal impact on viral fusogenicity or the efficacy of fusion inhibitors targeting HR1. Consequently, the HR1 and HR2 domains have been identified as main targets in the development of broad-spectrum HCoVs fusion inhibitors. Synthetic peptides, derived from the HR2 region, have been shown to competitively hinder the interaction between viral HR2 and the HR1 domain, thereby preventing the formation of viral 6-HB and hindering viral fusion and infection [133,134,135].

So, since the onset of the SARS-CoV-2 outbreak, it has been established that a peptide derived from the HR2 domain of the S2 subunit, named 2019-nCoV-HR2P, exhibits significant inhibitory activity against SARS-CoV-2 pseudovirus infection and S protein-mediated cell-to-cell fusion [125]. The fusion inhibitor targeting the HR2 domain (EK1) has been shown to disrupt viral 6-HB formation and inhibit viral fusion and entry (Figure 1B). Intranasal administration of EK1 to hACE2-transgenic mice has been shown to provide effective protection against SARS-CoV-2 infection by different SARS-CoV-2 VOCs, particularly the Omicron variant, which was resistant to most SARS-CoV-2 nAbs. EK1C4, a PEGylated, cholesterol-modified EK1 peptide, has been found to be able to inhibit the XBB sublineage of SARS-CoV-2 [123]. A similar peptide fusion inhibitor, P3, targeting HR1, including its modification P315V3, demonstrated the most efficient antiviral activity against SARS-CoV-2 variants and several other Sarbecoviruses, as well as other HCoVs. Furthermore, P315V3 demonstrated effective prophylactic efficacy against the SARS-CoV-2 Delta and Omicron variants in mice via intranasal administration [122]. Wang et al. has presented a self-assembling recombinant protein named HR1LS that contains three linked structural domains of the S2 subunit of the S protein: HR1, CH and SH domains. The construction was found capable of binding with HR2 domain of the S2 subunit of SARS-CoV-2 as well as other coronaviruses such as SARS-CoV, MERS-CoV, HCoV-229E, HCoV-NL63 and MjHKU4r-CoV-1 (a bat MERS-like coronavirus) thus showing itself as a potential entry and fusion inhibitor. Moreover, HR1LS combined with CF501 adjuvant induced the production of broad-spectrum nAbs against multiple variants of SARS-CoV-2 and other aforementioned coronaviruses in immunized BALB/c mice [126]. The other membrane fusion-inhibitory lipopeptide, designated IPB29, was also HR2-derived. In vitro experiments have previously shown that IPB29 effectively neutralizes two viral panels. Panel 1 included the previously circulated variants (D614G, Alpha, Beta, Gamma, Delta, Lamda and several mutants with specific substitutions (K417N, E484K, N501Y, P681R, N501Y/Δ69–70 and N501Y/P681H)). Panel 2 represented divergent Omicron sublineages, including the recently emerged BF.7. BQ.1.1, XBB, XBB.1.5, CH.1.1 and EG.5.1 variants. In a Syrian hamster model of SARS-CoV-2 infection, nebulized IPB29 has been shown to be highly effective against both the Delta and Omicron variants [124].

Based on previous studies exploring the potential of broad-neutralizing antibodies, a hypothesis was proposed that a multivalent S2 subunit vaccine, incorporating S2 from SARS-CoV, Bat-CoV RsSHC014 and SARS-CoV-2 S2mut, would provide extensive protection against human Betacoronaviruses [136,137]. As demonstrated in in vivo infection models, the vaccine in question was found to have a significant impact on virus titers in the lungs of immunized mice challenged with XBB, Pangolin-GD, BANAL-236, WIV1 and RsSHC014. It is interesting to note that the role of the effector T cells in S2-based protection is not pivotal. Virus titers in immunized mice challenged with mouse adapted SARS-CoV-2 virus (MA10) were similar to those in a group of mice preliminary treated with the anti-CD8 antibody. The vaccine-induced antibodies were shown to be non-neutralizing but broadly binding. Other findings of the study by P. J. Halfmann et.al. indicate that FcγR KO mice immunized with a multivalent S2 subunit vaccine exhibited a mean virus titer significantly higher than that observed in control group of immunized wild-type mice (mice were challenged with MA10). This observation suggests that antibody-dependent cellular effector mechanisms play a crucial role in the protection provided by immunization [136]. The S2-binding antibody, which was isolated from human B cells, binds to an epitope at the apex of S2 that is highly conserved among Betacoronaviruses. It was also non-neutralizing, but it was capable of inducing Fc-dependent antiviral responses in vitro, including antibody-dependent cellular cytotoxicity (ADCC) and antibody-dependent cellular phagocytosis (ADCP) [138].

Attempts to develop the pan-CoV vaccine or a universal cross-reactive vaccine antigen have been made since the very beginning of the pandemic. Many unique and original approaches have taken to create an effective vaccine. However, not all of them have made it to the world market or passed clinical trials. For example, X. Ma et al. designed several nanoparticle vaccines consisting of 24 copies of the RBD or RBD-HR protein subunit covalently conjugated to self-assembling non-haem ferritin from *H. pylori*. Both RBD and RBD-HR nanoparticles induced nAbs and promoted type-1 T-cell response in in vivo models and also provided protection in mice challenged with SARS-CoV-2. Notably, the conjunction of RBD or RBD-HR copies to ferritin increased the level of nAbs in animal sera compared to the monomeric protein subunit. RBD-HR, in particular, led to the production of broadly neutralizing antibodies that are capable of neutralizing other coronaviruses (SARS-CoV, MERS-CoV, HCoV-229E, HCoV-OC43 and RATG13) and demonstrated a stronger T-cell response in a BALB/c mice model [139]. Another example is the novel antibody HR-based vaccine designated HR121. HR121 is designed from the HR1–HR2–HR1 domains of the Wuhan-Hu-1 variant linked with peptides. HR121 has shown itself as a mimic of the native HR1 domain of the fusion-intermediate conformation of the S2 subunit and, thus, acted as a strong fusion inhibitor. It is important to note that HR121 exhibited broadly neutralizing activities against multiple variants of SARS-CoV-2 both in vitro and in vivo, especially against Omicron sublineages. HR121 provided long-term protection against SARS-CoV-2 Omicron infection after intranasal administration to Syrian golden hamsters [140,141]. A similar case is a vaccine candidate MSU-CoV-4/5 based on three SARS-CoV-2 antigen fragments (containing RBD and conserved regions of the S protein across SARS-related coronaviruses) and a novel adjuvant—spherical particles, originating from structurally modified tobacco mosaic virus. Despite the fact that the Wuhan-Hu-1 variant served as a matrix for recombinant antigens, sera obtained from animals vaccinated with MSU-CoV-4/5 has demonstrated broad neutralization activity against Omicron variants in vitro. MSU-CoV-4/5 has shown itself as a biologically safe vaccine candidate providing protection against WT SARS-CoV-2 in immunized golden Syrian hamsters [142,143]. Generally, the approach of utilizing highly conserved regions of the spike protein has once again proved itself promising as both HR121 and MSU-CoV4/5 are based on the WT SARS-CoV-2 variant; however, they provided strong protection against Omicron sublineages.

Given the continuous evolution of SARS-CoV-2, it is essential to develop novel and pragmatic vaccine design strategies to ensure long-term herd immunity and prevent the occurrence of a new pandemic or high-intensity epidemic. The use of pan-coronavirus vaccines is proposed to induce a cross-protective immune response against a range of Betacoronaviruses. However, developing such a universal vaccine is a significant challenge, given the RNA genomic structure of coronaviruses. This is especially true in light of the notable example of universal influenza vaccines (UIV), which have yet to be implemented in clinical practice [144]. As with UIV, selecting the appropriate target is essential. In the case of coronaviruses, the S2 subunit of the S protein could be an ideal candidate. This protein is a component of the immunogenic surface protein, which has a high degree of conservation [34,118,119,121,138,145]. Ideally, such a vaccine would provide long-lasting protection against SARS-CoV-2 as well as cross-protection against other coronaviruses.

## 5. Conclusions and Future Outlook

It has been over five years since the onset of the pandemic of SARS-CoV-2 that caused COVID-19, and the disease has now reached a low epidemic state [16]. The continuous evolution of SARS-CoV-2 has resulted in the emergence of a phylogeographically distinct clade of variants, designated Omicron, and these variants have become predominant. Despite its low pathogenicity, the infection still could be fatal, and this fact should be taken into consideration [146]. At present, there is an ongoing debate regarding the appropriate classification of the SARS-CoV-2 virus, specifically, whether serotypes should be assigned to it [147,148]. Moreover, the tools employed for the estimation of the direction of SARS-CoV-2 evolution are in a state of development [149,150,151]. The main feature of the evolution of SARS-CoV-2 was indicated as large “jumps” instead of gradual changing, which could be due to animal reservoirs, or a result of an evolution in immunocompromised individuals [152,153,154,155,156]. Considering these points, it is important to pay special attention to the vaccination of people with compromised health in order to limit the spread of the virus as well as monitoring the viral spread to the farm or domesticated animals.

The development of a pan-coronavirus vaccine could encompass broad-spectrum protection against the emerging viral variants. There are several strategies to achieve that goal. In order to strengthen the immune response to diversity of SARS-CoV-2 variants and related viruses, the development of a multivalent vaccine would require the inclusion of the spike protein (S protein) from several different viral strains [12,14,157,158]. The second approach that has received a spotlight in research is the use of heterologous booster vaccines. These vaccines have been shown to stimulate various branches of the immune response, including the broadening of T-cell responses [159,160,161,162]. The heterology of the vaccination regimen has the potential not only to involve the use of multiple vaccine platforms but also to use various methods for vaccine administration, such as the implementation of intranasal boosters [163]. These approaches were thoroughly reviewed by Saha et al. [164].

The current review, alongside the viral and vaccine coevolution, also discusses the possible use of the S2 subunit as a highly conserved region of the S protein for the development of a pan-coronavirus vaccine. It should be noted that the most widely used vaccines contain the full-length S protein, which includes the S2 subunit as well. However, previous research had shown that the levels of anti-S2 antibodies in vaccinated individuals were lower than in infected individuals or in individuals with hybrid immunity [165]. Additionally, the mRNA vaccine have been shown to induce a more robust response against the S2 subunit compared to vector-based vaccine [165,166]. This might be due to differences in the conformation of the S protein used in vaccine design and the conformation of the S protein present on the surface of intact and inactivated virions [167,168,169,170]. So, despite the presence of S2-subunits, the vaccine-induced response is probably shifted more towards anti-S1 epitopes. Despite the limited effectiveness of anti-S2 antibodies in neutralizing the virus, their broad cross-reactivity and capacity to trigger alternative antiviral defense mechanisms, such as antibody-dependent cellular cytotoxicity (ADCC), coupled with the significance of the S2 subunit in the viral architecture, make anti-S2 agents a promising candidate for developing a universal vaccine against all coronaviruses.

The limitation of this review is that we did not focus closely on the effectiveness of vaccine-induced cellular immunity against emergent viral variants, as this can be found in the review by J. Liu et al. [171].

## Data Availability

Not applicable.
